# AI-based predictive biomarkers for chronic neurological diseases: the rAIdD prospective, multicenter, observational study protocol

**DOI:** 10.3389/fneur.2026.1885919

**Published:** 2026-07-17

**Authors:** Simone Varrasi, Alfredo Pulvirenti, Vincenzo Catania, Maurizio Palesi, Concetto Spampinato, Davide Patti, Orazio Tomarchio, Giovanni Micale, Alessia Simone, Lisa Passarello, Federica Proietto Salanitri, Giovanni Patanè, Salvatore Ravidà, Clara Grazia Chisari, Giuseppe Zappalà, Emanuele D'Amico, Carlo Avolio, Federica Felicetti, Claudio Gasperini, Simone Rossi, Paolo Manganotti, Pierpaolo Busan, Carmelo Rodolico, Rossella Laudani, Roberto Marino, Massimo Villari, Francesco Patti, The rAIdD Study Group, Antonella Paola Agodi, Martina Barchitta, Daniela Giordano, Simone Palazzo, Aurora Zanghì, Carla Tortorella, Shalom Haggiag, Serena Ruggieri, Davide Dilena, Miloš Ajčević, Luca Gentile, Antonino Casile

**Affiliations:** 1 Department of Medical, Surgical Sciences and Advanced Technologies “G. F. Ingrassia”, University of Catania, Catania, Italy; 2 Department of Clinical and Experimental Medicine, University of Catania, Catania, Italy; 3 Department of Electrical, Electronic and Computer Engineering, University of Catania, Catania, Italy; 4 Department of Medical and Surgical Sciences, University of Foggia, Foggia, Italy; 5 Department of Neurosciences, University Neurology, Policlinico, Foggia, Italy; 6 Department of Neuroscience, San Camillo Forlanini Hospital, Rome, Italy; 7 Department of Medicine, Surgery and Neuroscience, University of Siena, Siena, Italy; 8 Department of Medical, Surgical and Health Sciences, University of Trieste, Trieste, Italy; 9 Department of Life Sciences, University of Trieste, Trieste, Italy; 10 Department of Clinical and Experimental Medicine, University of Messina, Messina, Italy; 11 Department of Engineering, University of Messina, Messina, Italy; 12 Department of Mathematical and Computer Sciences, Physical Sciences and Earth Science, University of Messina, Messina, Italy

**Keywords:** dementia, digital biomarkers, lifestyle, machine learning, Multiple Sclerosis, Parkinson's disease, risk factors, wearable devices

## Abstract

**Background:**

Chronic neurological disorders such as Multiple Sclerosis (MS), Parkinson's disease (PD), and Alzheimer's Disease (AD) represent a major global health burden characterized by progressive neurodegeneration, functional disability, and cognitive decline. Despite differences in etiology and clinical presentation, these conditions share multifactorial pathophysiological mechanisms influenced by genetic, environmental, and lifestyle-related factors. Advances in artificial intelligence (AI), wearable technologies, and multimodal clinical data integration offer new opportunities for identifying predictive digital biomarkers and improving personalized disease management. The rAIdD project (“*eHealth Network: AI and new ICT technology equipment for digital diagnosis*”) aims to develop an interoperable digital infrastructure to support early diagnosis, monitoring, and risk stratification in chronic neurological diseases. This study protocol describes the neurological component of the rAIdD network focusing on MS, PD, and AD.

**Methods and analysis:**

This prospective, multicenter, observational study involves six Italian academic and clinical centers and will enroll 780 participants: 300 MS, 150 PD, 150 AD, and 180 healthy controls. Participants will be followed for 18 months within a 48-month study period. Standardized clinical, neuropsychological, neuroimaging, and digital assessments will be performed at baseline and at 6-, 12-, and 18-month follow-ups. Clinical evaluation includes disease-specific disability and functional scales, mood and quality-of-life assessments, and lifestyle and environmental risk factor profiling. Continuous digital monitoring will be conducted using wearable sensors to collect biometric and behavioral data, including physical activity, sleep patterns, and cardiovascular parameters. Structural neuroimaging will be acquired longitudinally and integrated with clinical and digital data through a centralized web-based electronic data capture platform. Machine learning approaches will be applied to identify multimodal predictive biomarkers and model disease progression patterns across disorders.

**Ethics and dissemination:**

The study has been approved by the Ethics Committee of the coordinating center and by local ethics committees of all participating institutions. Written informed consent is obtained from all participants in accordance with the Declaration of Helsinki and the General Data Protection Regulation (GDPR 2016/679). Results will be disseminated through peer-reviewed publications, scientific conferences, and digital communication platforms to support knowledge translation and implementation of precision neurology approaches.

## 1 Introduction

Neurological disorders such as Multiple Sclerosis (MS), Parkinson's disease (PD), and Alzheimer's Disease (AD) represent a major and growing global health challenge, accounting for a substantial burden of disability, health care utilization, and socioeconomic cost (1). Although differing in pathophysiology and clinical expression, these disorders share a chronic, progressive course characterized by neuroinflammatory and neurodegenerative mechanisms that ultimately converge in cognitive and motor decline (2, 3). Their complexity calls for an integrated, multidisciplinary, and data-driven approach aimed at identifying shared mechanisms, modifiable risk factors, and predictive biomarkers that can guide personalized interventions.

This Study Protocol is part of the broader national research project rAIdD—“*eHealth Network: AI and new ICT technology equipment for digital diagnosis*”, funded by the Italian Ministry of Health under the Piano Operativo Salute–Traiettoria 2 (“*eHealth, advanced diagnostics, medical devices, and minimally invasive systems*”).

The overarching goal of the rAIdD initiative is to develop an interoperable digital infrastructure for the prevention, diagnosis, and management of high-impact chronic diseases through artificial intelligence (AI), big-data analytics, and ICT-based monitoring systems.

Within this national network, the neurological component specifically focuses on MS, PD, and AD, three paradigmatic chronic conditions that exemplify the interaction between neuroinflammation, neurodegeneration, and environmental and lifestyle-related risk factors.

Multiple Sclerosis is an immune-mediated inflammatory and neurodegenerative disease of the central nervous system (CNS), typically manifesting during early adulthood and predominantly affecting women, with a female-to-male ratio of approximately 2–3:1. It is the leading cause of non-traumatic disability in young adults (4, 5).

Parkinson's disease is a slowly progressive neurodegenerative disorder whose prevalence is estimated at 1–2% among individuals aged over 60 years and rises to 3–5% in those above 85 years (6).

Alzheimer's Disease accounts for more than half (7) of all cases of dementia, intended as a set of progressive neurocognitive syndromes. In Europe, AD prevalence exceeds 4% among individuals aged ≥ 65 years and increases to roughly 25% among those over 90 (8).

Although these diseases differ in etiology, clinical expression, and therapeutic options, they share a chronic, irreversible, and degenerative nature leading to both motor and cognitive impairment. Their pathogenesis is widely considered multifactorial, involving an interaction between genetic predisposition and environmental risk factors, except in the rare hereditary forms of PD and AD. Moreover, disease trajectories appear to be influenced by multiple behavioral and environmental determinants, which can modulate the rate of progression and clinical outcomes (4–6, 8).

In recent years, substantial attention has been directed toward the use of wearable sensors and remote-monitoring technologies for the continuous assessment of patients affected by chronic neurological diseases. Such systems provide valuable longitudinal data on physiological and behavioral parameters, thereby supporting the implementation of personalized and predictive medicine frameworks (9, 10). In this context, the CLADFIT-MS project has provided a relevant applied framework, combining patient-reported outcomes, clinical measures, and wearable-derived biometric data within a prospective, multicenter, real-world study design (11). Specifically, CLADFIT integrates digital measures such as physical activity, sleep patterns, and energy expenditure with standardized PRO instruments (e.g., MSIS-29, HADS, and PROMIS), enabling a multidimensional assessment of disease impact and treatment response in everyday clinical settings. Subsequent analyses have further supported the feasibility and clinical utility of this multimodal approach, highlighting the sensitivity of integrated digital and patient-reported metrics in capturing longitudinal changes in patients with multiple sclerosis (12).

The integration of neurophysiological and neuroimaging data with machine learning (ML) and artificial intelligence (AI) techniques has been shown to enhance diagnostic accuracy and enable earlier detection of subtle disease-related changes, supporting the development of digital biomarkers and individualized disease-monitoring tools (13). These computational approaches offer a scalable and data-driven method to improve clinical decision-making and optimize the management of high-impact neurological conditions (10).

Therefore, the establishment of large, structured clinical databases and interoperable digital infrastructures represents a key step toward integrating multimodal data and implementing advanced AI analytics. This integration holds the potential to accelerate progress in understanding disease mechanisms, improve early diagnosis, and design preventive and therapeutic strategies that address both disease onset and progression (14).

This model facilitates remote monitoring and follow-up, reducing patient burden—particularly in advanced disease stages—and optimizing healthcare resources by minimizing the need for routine in-person visits (15). In the long term, the integration of wearable technologies and telemedicine may support more continuous, patient-centered models of neurological care (16).

Within this general framework, the rAIdD study, particularly, aims to:

- Evaluate specific cognitive, motor, and functional profiles in MS, PD, and AD cohorts at baseline and over follow-ups;
- Identify common and disease-specific predictors of clinical progression using AI-based data integration of clinical, neurophysiological, and environmental variables.

By combining advanced AI techniques, standardized data collection, and longitudinal monitoring, this study will help clarify shared pathophysiological pathways across MS, PD, and AD. It will also provide clinicians and researchers with predictive models for disease progression and real-world patient stratification, ultimately contributing to precision neurology and to the goals of the rAIdD national network.

## 2 Methods and analysis

This is a prospective, multicenter, observational study involving the following Italian partners:

- University of Messina, Messina (administrative coordinating center)
- University of Catania, Catania (coordinating center of neurological network)
- University of Siena, Siena
- Hospital San Camillo Forlanini, Rome
- Hospital Ospedali Riuniti, Foggia
- University of Trieste, Trieste

The study duration will be 48 months (February 2023–February 2027), with a 18-month observation period for each enrolled participant. As the study is designed to (a) integrate multimodal data sources—clinical, neuroimaging, neurophysiological, and digital—and (b) apply advanced AI-based data analytics to identify biomarkers and predictors of disease progression, both a clinical research group and an ICT research group are involved.

### 2.1 Selection of participants and ethical considerations

A total of 600 patients will be enrolled (300 MS, 150 PD, and 150 AD), aged ≥ 18 years, recruited consecutively from outpatient and inpatient units at participating centers. *A priori* power analysis for a one-way ANOVA indicated that, with this sample size, the study has a statistical power of 80% (α = 0.05, two-tailed) to detect a small-to-medium effect size of Cohen's f = 0.13. This ensures the study is sufficiently powered to identify clinically significant differences among the three groups.

Inclusion criteria consist of a clinical diagnosis of:

- MS according to the 2017 McDonald criteria (2);
- Idiopathic PD according to the Movement Disorder Society (MDS) criteria, Hoehn & Yahr stage ≤ 3 (17);
- Alzheimer's disease dementia or prodromal MCI due to AD according to the NIA-AA criteria (18, 19);
- Disease duration ≤ 5 years from diagnosis;
- Ability to use (or to be assisted in using) digital monitoring tools (smart devices systems);
- Written informed consent obtained prior to participation.

Exclusion criteria, instead, include:

- Severe comorbid medical, psychiatric, or neurological conditions precluding study participation;
- Inability to provide informed consent;
- Contraindications to neuroimaging or digital monitoring.

In addition, 180 healthy subjects/controls will be recruited, corresponding to 30 participants at each of the six participating recruitment centers. At each center, healthy subjects/controls will be stratified into three age bands: 20–40 years, 40–60 years, and 60–80 years, with 10 participants recruited within each age band. Sex balance will be pursued within each age stratum, with a target allocation of 5 males and 5 females per age band. This stratification is intended to optimize age- and sex-matched comparisons across disease groups, whereby younger controls will serve as the reference group for participants with MS, while older controls will be compared with individuals with PD and AD. Healthy subjects/controls will undergo the same assessment battery as patients, thereby enabling robust normative benchmarking across digital, cognitive, and clinical measures.

Each participant will provide written informed consent in compliance with national and EU data protection regulations (GDPR 2016/679). General ethical approval for the project has been obtained by the coordinating center through the Local Ethical Committee of Messina (AOU “G. Martino” - AO “Papardo”–A.S.P.), with reference number 0165421, 27/12/2023. Each partner, then, obtained favorable ethical feedback from their related institution.

### 2.2 Data collection and measures

At baseline and at follow-up visits (6, 12, 18 months), participants will undergo standardized evaluations at their local centers. Such assessment will involve clinical, neuropsychological, digital, and neuroimaging measures.

#### 2.2.1 Clinical assessment

Core clinical measures include:

- For MS: Expanded Disability Status Scale [EDSS; (20)], disease duration, relapse rate, MRI lesion load, and brain atrophy;
- For PD: Movement Disorder Society Unified Parkinson's Disease Rating Scale [MDS-UPDRS; (21)], Hoehn & Yahr staging, and Non-Motor Symptoms Scale [NMSS; (22)], Activities of Daily Living and Instrumental Activities of Daily Living [ADL and IADL; (23, 24)] for the evaluation of autonomy;
- For Dementia: Activities of Daily Living and Instrumental Activities of Daily Living [ADL and IADL; (23, 24)] for the evaluation of autonomy, disease duration, electroencephalographic indicators;
- Cross-sectional health indicators related to lifestyle: this questionnaire has been specifically designed for this study for investigating the habits that could predict a better or worse outcome of the pathology over time. It consists of several sections investigating: general sociodemographic information, comorbidities, medications, cardiovascular risk factors, social contacts, quality of living environment, diet, vaccination status, number of general anesthesia, sun exposure, physical activity, smoking/alcohol/caffeine use, and sleep habits. Continuous lifestyle monitoring will be also supported by wearable devices providing real-time physiological and activity data (see 2.2.3 paragraph).

#### 2.2.2 Neuropsychological assessment

The neuropsychological assessment will be carried out both at baseline and at follow-up visits, and will include:

For MS:

- The Brief International Cognitive Assessment for Multiple Sclerosis [BICAMS, (25)]. This tool has been recommended as an international measure requiring approximately 15 min. It provides a screening evaluation that, despite its brevity, is capable of detecting potential cognitive deficits. In the present study, the version validated on the Italian population will be used (26). BICAMS is composed of: the Symbol Digit Modalities Test (SDMT; spoken response), the first five learning trials of the California Verbal Learning Test (CVLT-II) and the first three learning trials of the Brief Visuospatial Memory Test-Revised [BVMT-R, (27)]. The Symbol Digit Modalities Test assesses sustained attention, working memory, and information processing speed. The SDMT is highly sensitive to cognitive impairment associated with MS and is now widely recognized as a measure for rapid cognitive screening (28). The first five trials of CVLT-II assess verbal attention span, verbal learning memory, and the use of learning strategies (29). The first three learning trials of BVMT-R evaluate visual-spatial memory but also constructive praxis; it includes measures of omission, distortion, perseveration, rotation, misplacement, and size errors of perceptual target stimuli (30).
- The Beck Depression Inventory-II [BDI-II, (31)] for the evaluation of depressive symptoms. Depression is the most frequent mood disorder in MS, the prevalence is approximately 30.5% for depression and 22.1% for anxiety (32, 33). The BDI-II demonstrates strong psychometric reliability for assessing the severity of depressive symptoms in individuals with MS and has been validated for this purpose (34).
- Fatigue Severity Scale [FSS, (35)], assessing the impact of fatigue on daily functioning. Fatigue represents a prevalent symptom in Multiple Sclerosis, manifesting as a continual sense of exhaustion that significantly disrupts everyday activities. According to a systematic review (36) among populations not limited to Clinically Isolated Syndrome or non-disabled patients, fatigue prevalence ranged from 36.5% to 78.0%. Across adult studies, independent of MS subtype, disability, or assessment method, prevalence ranged from 18.2% to 97.0%.
- Multiple Sclerosis Quality of Life-29 (37). Multiple sclerosis may have a significant impact on perceived quality of life, and the MSQOL-54 is the most widely used questionnaire for investigating this aspect. MSQOL-29 is a shortened version of the instrument, which proves to be more versatile in certain clinical settings. The questionnaire items belong to the following thematic areas: physical function, bodily pain, sexual function, emotional wellbeing, cognitive function, health distress, social function, health perceptions, overall quality of life, change in health (38).

For PD:

- Mini-Mental State Examination [MMSE, (39)] for measuring general cognitive functioning. MMSE is likely the most commonly used screening instrument for detecting cognitive deterioration. It has good and widely validated effectiveness in measuring the severity of moderate and severe cognitive decline. It will be used a validated Italian version of MMSE (40). The test consists of sub-items that briefly assess spatial and temporal orientation, immediate and delayed memory, naming and repetition abilities, calculation and working memory, constructive praxis, command/ instruction execution, and writing.
- Montreal Cognitive Assessment [MoCA, (41)] for the assessment of mild to moderate cognitive deficits. Correlation between MoCA and MMSE scores indicates a significant but moderate correlation between the two instruments, consistent with the idea that they evaluate partially different aspects of cognitive functioning. As a screening tool, MoCA is more sensitive to mild-to-moderate decline and it includes items aimed at assessing visuospatial and executive functioning.
- Frontal Assessment Battery [FAB, (42)] for the evaluation of executive functions. Substantial evidence indicates the presence of executive deficits in Parkinson's disease, with the most pronounced impairments observed in working memory, inhibitory control, and concept formation (43). This instrument requires approximately 10 min to administer and evaluates six subdomains: conceptualization, lexical fluency, mental flexibility, motor programming and planning, sensitivity to interference and inhibitory control, and prehension behavior.
- Beck Depression Inventory-II [BDI-II, (31)] evaluating depressive symptoms. The prevalence of depression in Parkinson's disease ranges from 17% to 38%. The pattern of mood disturbance most frequently observed in this condition includes guilt-related thoughts, asthenia, apathy and suicidal ideation. The Italian version of the BDI-II has been shown to possess good diagnostic sensitivity for depressive states in Parkinson's disease (44).

For dementia:

- Frontal Assessment Battery [FAB, (42)], assessing executive functions. Their impairment can be observed in multiple forms of dementia, including Lewy Body Dementia, Vascular Dementia, Parkinson's disease Dementia, the frontal variant of Alzheimer's Disease, Frontotemporal Dementia, and Progressive Supranuclear Palsy. Different patterns of impairment are observed according to the neurodegenerative variant (45).
- Mini-Mental State Examination [MMSE, (39, 40)], for evaluation of cognitive general functioning, as described above.
- Montreal Cognitive Assessment [MoCA, (41)], for measuring mild to moderate cognitive deficits, as discussed above.

#### 2.2.3 Digital assessment

The digital assessment will be aimed at continuous, objective, and multimodal measurement that will complement the clinical and neuropsychological examinations performed throughout the study. Its objective is to capture subtle changes on an individual level over time and define potential digital biomarkers of the participants' clinical trajectories.

Digital data collection is based on two leading sources, namely: i) wearable sensors, allowing for remote, passive, and longitudinal monitoring of daily activity, sleep patterns, and cardiovascular dynamics; and ii) magnetic resonance neuroimaging, providing both structural and functional brain measures at preselected points in time. Together, these data streams support a multimodal profiling approach, enabling a more comprehensive evaluation of status and progression in participants.

The digital assessment is expected to result in a set of quantitative indicators, including measures of physical activity, quality of sleep, heart rate and variability, and neuroimaging-derived indices of brain structure and connectivity. These will contribute to the longitudinal dataset used for statistical and machine-learning analyses (see Section 2.4), allowing the study of multi-modal associations and predictive patterns. Overall, this digital component increases the sensitivity of the study by providing real-world behavioral patterns and neurobiological features that might remain concealed when using more traditional assessments.

##### 2.2.3.1 Wearable devices

Physiological parameters will be monitored over a standardized 5-day period at each scheduled assessment timepoint, namely at baseline and at the 6-, 12-, and 18-month follow-up visits, using Fitbit wearable devices. Whenever feasible, the 5-day monitoring window will be scheduled to include both weekdays and weekend days. Thus, wearable-derived biometric and behavioral data will be collected repeatedly across the study, allowing the assessment of both between-group differences and within-participant changes over time.

The 5-day monitoring window was selected as a pragmatic compromise between ecological validity, participant burden, device feasibility, and standardized implementation across centers. This window is intended to provide a harmonized short-term estimate of habitual physical activity, sleep patterns, heart rate, and related physiological parameters at each study visit. Although it does not aim to capture the full range of intra-individual variability, the repeated acquisition of 5-day monitoring windows at each timepoint will enable longitudinal modeling of changes in digital biomarkers over the 18-month observation period. Participants will be instructed to wear the device continuously during each monitoring window, and wearable-derived variables will be summarized using aggregated daily and temporal indicators across valid monitoring days.

The selected model is the Fitbit Inspire 3 (39.32 mm x 18.6 mm, 11.75 mm thick, and 17.69 grams), powered by a lithium-polymer battery with a battery life of 10 days, a full charge in 2 h, a Bluetooth radio transmitter and firmware version 20001.214.24 and Fitbit app version 4.21, kept constant over the full study period. The device records movement data with a temporal resolution of 1 min and stores these data locally for up to 7 days without synchronization. Heart rate data are sampled at 1-s intervals during physical activity and at 5-s intervals during routine conditions. In addition, the device retains aggregated daily metrics for up to 30 days. The device has an operating temperature of 0–40 °C, features an AMOLED Corning^®^ Gorilla^®^ Glass 3 display, and is water resistant up to 50 meters. Finally, it is equipped with a 3-axis accelerometer, an optical heart rate sensor, a vibration motor, red and infrared sensors for measuring oxygen saturation, an ambient light sensor, and a temperature sensor (46).

For every participant, a dedicated Gmail account will be generated, structured using only the patient ID to avoid linking identifiable information to the collected data. The account will be used only for creating and setting up the Fitbit device, accessing the app, and synchronizing data for the whole monitoring period.

Device assignment requires preliminary verification of the patient's smartphone technical prerequisites: minimum requirements Android 11.0 and iOS 16.4, WiFi connectivity, and Bluetooth LE compatibility. The clinician will configure the device, logging in via the dedicated account, and disabling all the wrist notifications in order to avoid the transmission of personal information. At the moment of delivery, the patient will be instructed to keep synchronization active via the Fitbit app for the whole duration of the monitoring.

After the observation period, when returning the device, the final synchronization takes place, and data will be exported in compressed format (.zip). The data shared with the technical team consist exclusively of anonymized physiological parameters, with no direct identifying reference.

At this stage, an assessment of usability and acceptability of the wearable equipment will be conducted via the System Usability Scale [SUS, (47)] and the Unified Theory of Acceptance and Use of Technology (48), both administered to the participant when the Fitbit is returned. The SUS is a self-report questionnaire consisting of 10 items designed to assess perceived system usability. Items are rated on a 5-point Likert scale ranging from strongly disagree to strongly agree, with alternating positively and negatively worded statements. The scale yields a single underlying factor reflecting overall usability, and a total score converted to a 0–100 range. UTAUT is a multi-item self-report instrument used to assess technology acceptance and use. Items are rated on Likert-type scales (typically 5 or 7 points) and load on four core latent factors: Performance Expectancy, Effort Expectancy, Social Influence, and Facilitating Conditions. Additional items assess Behavioral Intention and Use Behavior.

Fitbit raw data exported in compressed format will be remotely stored in dedicated servers handled by the centers involved in the study. On each server, raw data will be cleaned and filtered, in order to extract the following candidate digital biomarkers, organized into three complementary data streams:

a) Daily aggregated metrics, which include:

- Step count, distance covered, calories burned, activity-level minutes categorized as sedentary, lightly active, moderately active, and very active, all derived from the device's 3-axis accelerometer;
- Resting heart rate, time spent and calories burned in each heart rate zone (below fat burn, fat burn, cardio, and peak) and VO_2_ max estimated via Fitbit's proprietary demographic algorithm, all derived from the photoplethysmographic (PPG) optical sensor.

b) Temporal metrics at 1-min time resolution, which include:

- Per-minute step count, distance, calories, heart rate, heart rate zone classification and heart rate variability (HRV) derived from the PPG sensor;
- Activity level classification produced by a proprietary fusion algorithm combining accelerometry and PPG data.

c) Sleep metrics at 1-min time resolution, which include:

- Duration and episode counts for light, deep, and REM sleep phases, total time in bed, sleep efficiency, minutes to fall asleep, minutes awake after sleep onset, and per-phase respiratory rate, which are generated by a proprietary multi-sensor fusion algorithm integrating accelerometry and PPG data;
- Peripheral oxygen saturation (SpO_2_) collected via the device's red and infrared PPG sensors;
- Nightly skin temperature deviation from baseline, recorded by the wrist-based temperature sensor.

It should be noted that all derived metrics (sleep staging, activity classification, VO_2_ max, SpO_2_, respiratory rate, and HRV) are computed by Fitbit's proprietary algorithms rather than constituting raw sensor signals; accordingly, these variables will be treated as consumer-grade digital biomarker proxies and interpreted in the context of their known measurement limitations. Importantly, the Fitbit Web API does not provide access to raw accelerometer or PPG waveform data: all fields available for export are already processed outputs of Fitbit's on-device and cloud-based algorithms. A feature extraction step from raw sensor signals is therefore not applicable in this context; instead, the exported summary metrics themselves constitute the digital features for the analysis. A complete list of all fields extracted from the Fitbit Web API, organized by data stream (Daily, Temporal, and Sleep), together with a description of each field and its unit of measurement, is provided in Supplementary File 1.

Finally, Fitbit data processed by each center will be integrated and stored in a central database, which is integrated within the ICT-managed digital infrastructure (see Section 2.3).

Following data export and device return procedures, the dedicated Gmail account will be disconnected from the participant's smartphone and the Fitbit application will be uninstalled. This step guarantees the definitive interruption of data synchronization and further strengthens data protection by eliminating any potential residual monitoring or data transfer in accordance with GDPR data minimization principles.

**Figure 1:**
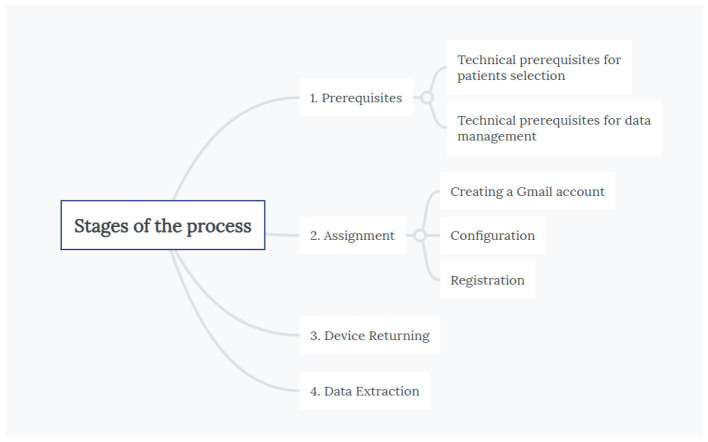
Stages of wearable equipment management.

The overall procedure is represented in Figure 1. The whole process has been designed to be fully compliant with the principles of GDPR, using data minimization and pseudonymization with access control. Only the clinician is able to trace the ID used for managing the device to the patient's identity; no member of the technical or research team has the capability to do so. Structured account management, end-of-use deletion, and secure storage on a private cloud infrastructure ensure a high level of data confidentiality. All data collection, transfer, and analysis procedures are conducted in a secure and compliant manner.

A dedicated tool has been developed to facilitate a simple and comprehensive analysis of biomedical data.

**Figure 2:**
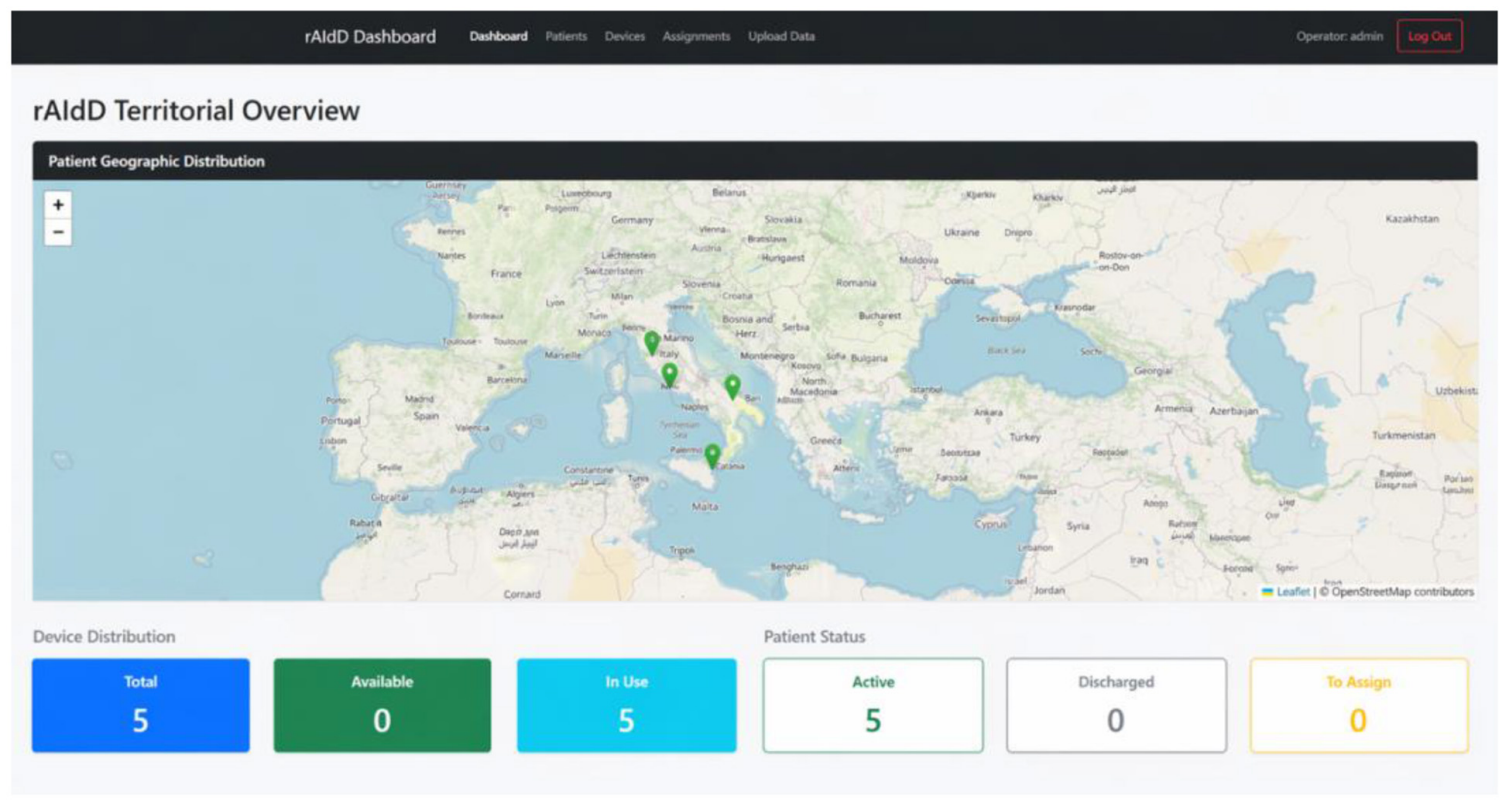
Regional overview of the biomedical device management dashboard.

Figure 2 shows the dashboard's home screen, which features a geographical map allowing users to locate devices currently in use, along with information on the number of active and in-use devices and their distribution by region.

**Figure 3:**
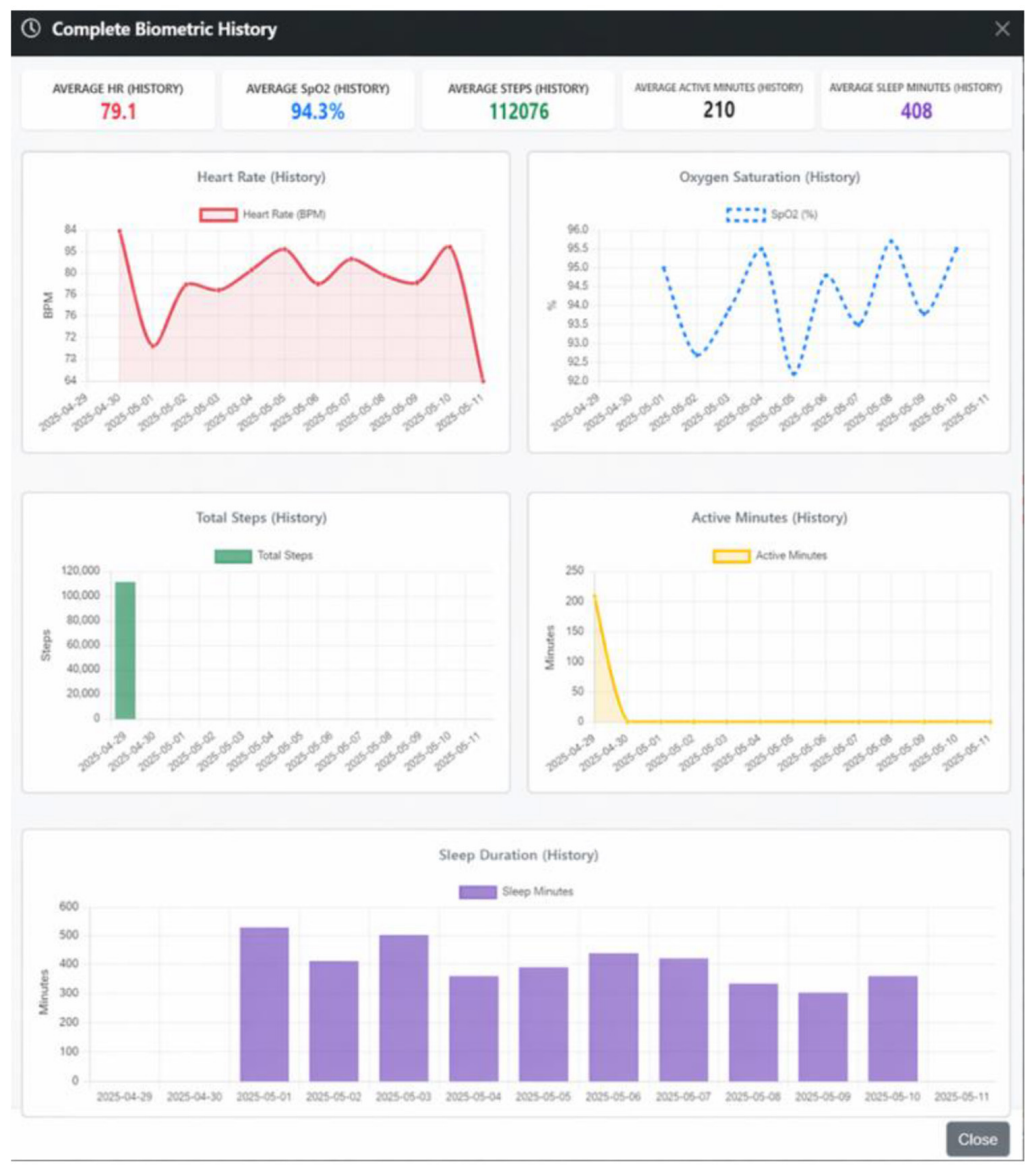
Physiological parameters history.

The second tab of the dashboard is dedicated to patient registration, keeping personal and sensitive data anonymous, whilst the third is dedicated to the registration of the wearable devices provided. Finally, in the last sections, it is possible to:

- manage the assignment and return of the device to each patient

- view the history of biomedical data in the form of a line graph and the averages of the main data (Figure 3).

##### 2.2.3.2 Neuroimaging

The imaging protocol includes multi-modal structural sequences designed to detect morphological changes and white matter hyperintensities. The acquisition will follow a longitudinal design with scans scheduled at six-month intervals.

The raw data, originally generated in DICOM format and stored on physical media, will first be copied to a secure archive. Subsequently, the DICOM files will be converted into the research-standard NIfTI format and organized according to the Brain Imaging Data Structure (BIDS) standard.

To comply with privacy regulations and mirror the security measures of the wearable module, a strict two-step de-identification process is applied. First, Protected Health Information (PHI) is stripped from all image metadata. Second, a defacing algorithm is applied to the high-resolution 3D T1 images to remove facial features, ensuring that 3D surface reconstruction cannot be used to re-identify the subject.

Original identifiers are replaced by a pseudo-anonymized research code, with the linkage key stored in a separate, secure relational database accessible only to the principal investigator. The processed, anonymized neuroimaging data will be uploaded to the central research repository before being integrated into the central database alongside the biometric wearable data.

### 2.3 Digital infrastructure and web platform

To support the standardized collection, storage, and management of clinical data across all participating centers, a dedicated web-based platform has been developed as part of the rAIdD digital infrastructure. This platform serves as the central electronic data capture (EDC) system for the neurological component of the study, enabling secure, multi-center data entry while ensuring compliance with data protection regulations and facilitating longitudinal patient monitoring.

#### 2.3.1 System architecture and technology stack

The web platform follows a modern full-stack architecture built on the Remix framework (version 2.1), a React-based server-side rendering framework that provides optimized performance and enhanced security through server-side data handling. The frontend interface is developed using React 18 with TypeScript, ensuring type safety and maintainability, while the styling layer employs TailwindCSS with Radix UI components to deliver a responsive, accessible, and consistent user experience across devices.

The backend infrastructure relies on Node.js as the runtime environment, with MongoDB 6.0 serving as the primary database management system. MongoDB was selected for its document-oriented data model, which accommodates the flexible and hierarchical structure of clinical questionnaires and assessment data. The database is configured with replica set functionality to ensure data redundancy and high availability. Prisma ORM (version 6.12) provides a type-safe database access layer, enabling efficient query construction and data validation.

The application is containerized using Docker, facilitating consistent deployment across different environments and simplifying infrastructure management at each participating site. This containerization approach also supports future scalability requirements as the study progresses and data volume increases.

#### 2.3.2 Core functional modules

The platform is organized into several interconnected modules designed to support the complete data collection workflow of the study:

- Patient Registry Module. This module manages participant enrollment and demographic data collection. Each patient record captures personal information (name, date of birth, gender, contact details), institutional affiliation, and primary pathology classification (Multiple Sclerosis, Parkinson's Disease, Alzheimer's Disease, or Healthy Control). The system supports the multi-center study design by allowing data entry from all six participating sites (Catania, Messina, Foggia, Roma, Siena, and Trieste) while maintaining centralized data aggregation.
- Clinical and Neuropsychological Assessment Module. The platform implements a comprehensive electronic case report form structured into disease-specific sections. For neurological assessments, the system supports standardized data entry for:
° Multiple Sclerosis: EDSS scoring with individual functional system subscores (pyramidal, cerebellar, brainstem, sensory, bowel/bladder, visual, and mental functions), BICAMS battery (CVLT-II, SDMT, BVMT-R), FSS fatigue assessment, MSQOL-29 quality of life measures.
° Parkinson's Disease: MDS-UPDRS scores, Hoehn & Yahr staging, MoCA, FAB, ADL/IADL functional scales.
° Dementia: MMSE, MoCA, FAB, ADL/IADL assessments.
The assessment module employs a tabbed interface that organizes data by evaluation timepoint (baseline, 6-month, 12-month, and 18-month follow-ups), enabling longitudinal tracking of clinical progression. Each section supports various input types including numeric values, dates, categorical selections, and conditional nested questions that appear based on prior responses.
- Mood Assessment Module. Depression screening is implemented through the Beck Depression Inventory-II (BDI-II), with dedicated data entry fields integrated within the disease-specific assessment sections. The platform stores raw item responses and enables downstream score calculation for analysis purposes.
- Wearable Device Management Module. A dedicated module supports the management of Fitbit devices used for continuous biometric monitoring. This module enables:
° Registration of devices in the study inventory with unique device identifiers;
° Assignment of devices to enrolled participants with specified monitoring periods (start and end dates);
° Tracking of device assignment status (active or completed);
° Maintenance of complete assignment history for audit and traceability purposes.
This tracking system ensures that each wearable device can be accurately linked to participant data while maintaining separation between device management and personal identifiers, supporting the pseudonymization approach described in Section 2.2.3.1.
- Technology Acceptance Assessment Module. Given the study's focus on digital monitoring tools, the platform includes dedicated modules for assessing participant acceptance and usability perception of wearable devices:
° System Usability Scale (SUS): A 10-item questionnaire implemented with 5-point Likert scales. The platform automatically calculates the standardized SUS score (0–100 range) using the established scoring algorithm.
° UTAUT Questionnaire: A comprehensive 31-item assessment organized into eight theoretical constructs (Performance Expectancy, Effort Expectancy, Attitude, Social Influence, Facilitating Conditions, Self-Efficacy, Anxiety, and Behavioral Intention). The platform computes mean scores for each construct and an overall acceptance index.
- Document Management Module. The platform provides secure file upload capabilities for storing patient-related documentation, including clinical reports and imaging results. Files are stored with cryptographic hash-based naming to prevent unauthorized access through filename guessing, and all uploads are linked to specific patient records for organizational integrity.

#### 2.3.3 Data security and privacy compliance

The platform implements multiple layers of security to ensure compliance with GDPR (2016/679) and Italian data protection regulations:

- Authentication and Access Control. User authentication is enforced through session-based mechanisms with secure HTTP-only cookies. Passwords are stored using bcrypt hashing with appropriate computational cost factors. All protected routes require valid authentication before granting access to patient data.
- Data Pseudonymization. The system architecture enforces separation between identifying information and clinical data. Patient identifiers used within the platform are internal database references that cannot be linked to real-world identities without access to the controlled mapping maintained exclusively by authorized clinical personnel at each site.
- Secure Data Transmission. The platform is designed for deployment with HTTPS encryption, ensuring that all data transmitted between client browsers and the server is protected against interception.
- Audit Capabilities. Database records maintain timestamp fields (createdAt, updatedAt) that enable tracking of data entry and modification events, supporting data integrity verification and regulatory compliance requirements.

#### 2.3.4 Data integration and interoperability

The platform serves as the central repository for clinical and questionnaire data, which will be integrated with biometric data from wearable devices and neuroimaging data from MRI acquisitions for the AI-based analytics described in Section 2.5. The MongoDB document model facilitates flexible data structures that can accommodate the heterogeneous nature of multimodal clinical research data.

Processed biometric data from Fitbit devices, following the cleaning and extraction procedures performed at each center (as described in Section 2.2.3.1), will be uploaded to the central database and linked to corresponding patient records through the device assignment tracking system. This integration enables the correlation of continuously monitored physiological parameters with discrete clinical assessments captured through the platform.

#### 2.3.5 User interface and clinical workflow support

The platform interface has been designed to support efficient clinical workflows while minimizing data entry burden. Key usability features include:

- A responsive dashboard providing summary statistics on enrolled participants, completion status, and distribution across sites and disease categories;
- Advanced search and filtering capabilities enabling rapid identification of specific patient records;
- Sortable data tables with multi-field search functionality;
- Visual indicators distinguishing complete from incomplete assessments;
- Tabbed navigation for multi-section questionnaires reducing cognitive load during data entry;
- Confirmation dialogs for irreversible actions to prevent accidental data loss.

The platform supports both light and dark display themes to accommodate different clinical environments and user preferences, and the interface is optimized for use on standard desktop displays typically available in clinical settings.

In summary, the rAIdD web platform provides a robust, secure, and user-friendly digital infrastructure for multi-center clinical data collection. Its modular architecture supports the comprehensive assessment battery required by the study protocol while maintaining the flexibility necessary to accommodate the distinct evaluation requirements of MS, PD, and AD cohorts. The integration capabilities of the platform position it as the foundation for the multimodal data analytics that represent a core objective of the rAIdD initiative.

### 2.4 Data analysis

The analytical strategy of the rAIdD neurological study aims to identify multimodal predictive biomarkers of disease progression across MS, PD and AD by integrating wearable-derived digital metrics, lifestyle and clinical variables, and neuroimaging data. Analyses will be performed on both baseline and longitudinal follow-up data (6, 12, and 18 months), enabling the assessment of both between-group differences and within-participant changes over time. The analytical pipeline includes the following stages:

1) Feature and outcome definitions
2) Temporal alignment of features
3) Feature selection
4) Multimodal integration model

**Figure 4:**
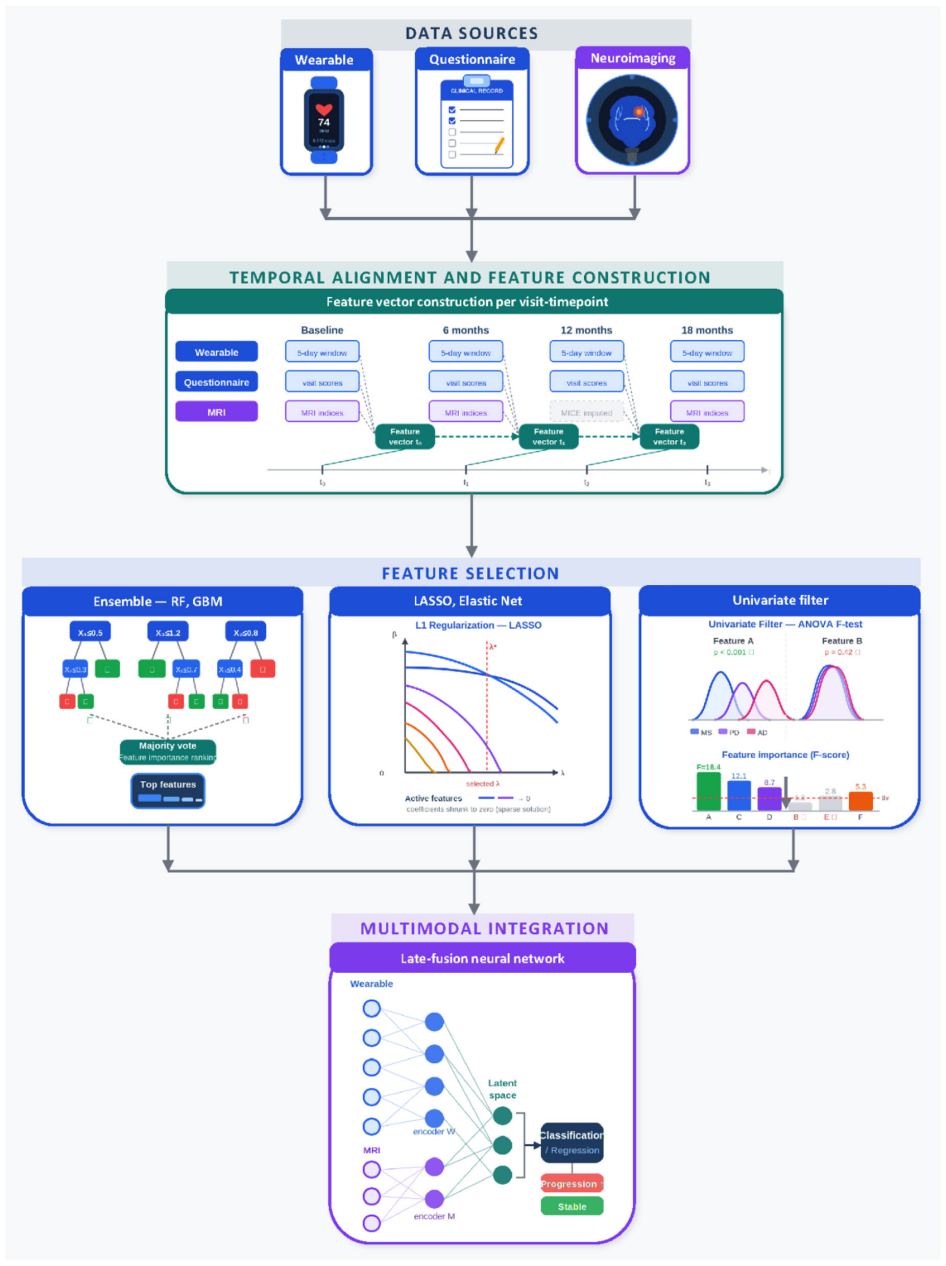
Workflow diagram of the analytical pipeline.

A workflow diagram of the analytical pipeline is depicted in Figure 4. In the next subsections, we detail each stage of the analysis.

#### 2.4.1 Feature definition and outcome specification

The candidate features entering the analytical pipeline comprise three complementary sets of predictor variables. The first set consists of wearable-derived digital metrics (Daily, Temporal, and Sleep signals extracted from the Fitbit Inspire 3; see Section 2.2.3.1). The second set consists of lifestyle and clinical variables derived from the general questionnaire administered to all participants at each visit, including: dietary habits (Mediterranean diet adherence items), physical activity self-report (days of vigorous and moderate activity, daily sedentary minutes), sleep duration, substance use (smoking, alcohol, caffeine), comorbidities, and current medication use. The third set consists of neuroimaging-derived morphometric and structural connectivity indices extracted from MRI acquisitions performed at each visit. Sociodemographic variables (sex, age, level of education, living situation, occupational type) will be included as covariates in all models. Disease-specific neurological scale scores (EDSS and functional system subscores for MS; UPDRS and Hoehn & Yahr stage for PD; MMSE, MoCA, FAB, ADL, and IADL for AD) will serve as the primary outcomes and progression targets of the models, rather than as input features, in order to avoid circularity. Composite cognitive scores from the BICAMS battery (CVLT-II, SDMT, BVMT-R), the FSS fatigue scale, disease-specific quality-of-life measures (MSQOL-29), and MRI-derived lesion counts (T1 and T2) will likewise be treated as outcome variables. This feature-outcome separation ensures that the models are trained to predict clinically meaningful endpoints from independent predictor signals.

Study endpoints are organized into three tiers. The primary endpoints are the predictions of disease progression at different timepoints, operationalized as the change from baseline in the primary disease-specific clinical scale for each condition: EDSS change for MS, UPDRS total score change for PD, and MMSE change for AD. These endpoints directly reflect the core aim of the study: identifying multimodal digital biomarkers that can track neurological deterioration over time. The secondary endpoints include the prediction of change in more granular functional and cognitive measures: for MS, variation in BICAMS subscores (CVLT-II, SDMT, BVMT-R), FSS fatigue score, MSQOL-29 composite scores, and MRI lesion counts (T2 and T1); for PD, variation in Hoehn & Yahr stage, MoCA, FAB, ADL, and IADL; for AD, variation in MoCA, FAB, ADL, and IADL. The exploratory endpoints are: (i) binary classification of patients vs. healthy controls; (ii) multi-class disease discrimination (MS vs. PD vs. AD); and (iii) unsupervised identification of patient subgroups with homogeneous progression trajectories via clustering. All endpoint analyses will be supervised by the clinical scales listed above, which serve exclusively as outcome variables and are never used as model input features.

#### 2.4.2 Temporal alignment and feature construction

Prior to any modeling, a temporal alignment and feature-construction step will be applied to reconcile the three data streams: wearable data over a 5-day window at each visit, neuroimaging at each 6-month timepoint, and clinical and neuropsychological assessments at each scheduled visit. The unit of analysis is the visit-timepoint, defined as the set of assessments scheduled for a given participant at baseline, 6, 12, or 18 months. For each visit-timepoint, a single multimodal feature vector will be constructed as follows. Wearable-derived variables (Daily, Temporal, and Sleep metrics) will be summarized into scalar summary statistics computed across valid monitoring days within the 5-day window associated with that visit (e.g., mean, standard deviation, and interquartile range of each daily metric; aggregated temporal statistics such as peak and nocturnal mean heart rate). These scalar summaries constitute the wearable feature block for that timepoint. Neuroimaging-derived morphometric and connectivity indices will be extracted from the MRI acquisition performed at the same visit. Where MRI and wearable monitoring windows do not coincide within a ±14-day tolerance, that modality will be treated as missing for that timepoint. Clinical and neuropsychological scores recorded at the visit will be split according to their analytical role: lifestyle and clinical variables from the general questionnaire (see above) will be aligned to the same timepoint as input features, while neurological scale scores and neuroimaging-derived indices will be aligned as outcome variables. When a modality is missing at a given timepoint (due to device non-wear, image acquisition failure, or scheduling deviation), multiple imputation by chained equations (MICE) will be applied, with missingness mechanism assessed via Little's MCAR test. The resulting aligned, timepoint-level feature matrix will serve as input to both the feature-selection and multimodal integration stages. For longitudinal progression modeling, feature vectors from successive timepoints will be stacked to form individual trajectories, enabling the model to capture within-participant changes over the 18-month observation period.

#### 2.4.3 Feature selection

Three complementary model classes will be applied to the full set of multimodal candidate features derived from all three data sources to ensure that the most informative predictors are identified across the entire feature space, regardless of their modality of origin: (i) tree-based ensemble methods (Random Forest and Gradient Boosting), using mean decrease in impurity and permutation importance as feature-importance criteria; (ii) regularized linear models (LASSO and Elastic Net), where the magnitude of non-zero regression coefficients after L1 penalization serves as the selection criterion; and (iii) a univariate filter step (ANOVA F-statistic or Kruskal–Wallis test, depending on the distributional properties of each variable) applied prior to the wrapper methods to remove clearly uninformative features. All feature-selection models will be evaluated under a repeated stratified k-fold cross-validation scheme, with stratification by disease, sex and age. Model performance will be assessed using the area under the receiver operating characteristic curve (AUC-ROC) for group discrimination and the mean absolute error (MAE) for continuous progression outcomes. Feature stability across folds will be quantified using the Jaccard similarity index on the top-ranked feature sets, and only features ranking consistently across folds will be retained as candidate biomarkers for the integration stage.

#### 2.4.4 Multimodal integration

The features retained from the selection stage will be integrated using a multimodal neural network architecture. The intended architecture follows a late-fusion design. Modality-specific encoder branches (one fully connected sub-network per data modality) independently project each input domain into a shared latent representation. Branches are then concatenated and passed through a joint classification or regression head. This design is preferred over early fusion given the heterogeneous dimensionality and temporal structure of the two data streams. The network will be trained using cross-entropy loss for disease-group classification tasks and mean squared error for continuous progression modeling, with dropout regularization and batch normalization applied within each encoder branch to reduce overfitting. Hyperparameter optimization (learning rate, dropout rate, layer depth, and latent dimension size) will be performed via Bayesian optimization on the validation fold. The multimodal model will be validated using the same repeated stratified k-fold cross-validation scheme described above, with model performance reported as AUC-ROC, sensitivity, specificity, and F1-score. Given the sample size, an independent hold-out test set will be defined prior to any model training and used exclusively for final performance reporting, to provide an unbiased estimate of generalization.

### 2.5 Study timing

In Table 1 is shown the schedule of the study organized by months (M).

**Table 1:** Schedule of the study by months.

Activity	M1-M6	M7-M12	M13-M18	M19-M24	M25-M30	M31-M36	M37-M42	M43-M48
Design and management of ICT infrastructure								
Review of data in the literature								
Pilot study								
Enrolment								
Clinical evaluation								
Collection of data on lifestyle risk factors								
RMN imaging								
Collection of data with wearable devices								
Evaluation of the pilot study								
Statistical analyses								
Dissemination								

Grey color indicates in which months the activities listed on the left will take place.

## 3 Discussion

The rAIdD neurological study is designed to address a critical gap in contemporary neurology: the need for scalable, longitudinal, and integrative approaches capable of capturing disease trajectories in chronic neurological disorders. By combining standardized clinical and neuropsychological assessments with continuous digital monitoring and advanced artificial intelligence (AI)-based analytics, this protocol proposes a comprehensive framework for the identification of predictive biomarkers across Multiple Sclerosis, Parkinson's disease, and Alzheimer's Disease.

A major strength of the present study lies in its cross-disease design. Although MS, PD, and AD differ substantially in etiology and clinical presentation, they share convergent pathways of neurodegeneration, progressive disability, and cognitive decline, making cross-comparative designs conceptually and clinically valuable for understanding shared and distinct disease mechanisms.

The integration of wearable-derived digital biomarkers represents a further innovative aspect of this protocol. Wearable sensors combined with computational analysis have demonstrated potential in capturing real-world motor and non-motor features relevant to PD and other neurodegenerative diseases. For example, gait and mobility derived from wearable sensors have been shown to predict PD progression and correlate with clinical measures in prospective cohorts, supporting digital biomarkers as quantifiable predictors of disease trajectory in PD populations (49, 50).

Additionally, systematic reviews indicate that wearable sensors, when paired with machine learning classification, have the potential to improve diagnostic accuracy and continuous monitoring in PD relative to traditional clinical assessment alone (51, 52).

Neuroimaging data, acquired within a harmonized and longitudinal framework, add a complementary neurobiological dimension to the study. Structural MRI features have been widely used in clinical research to characterize brain atrophy and white matter changes associated with Alzheimer's disease and related dementias. Recent narrative reviews indicate that AI-based analysis of neuroimaging data can improve early diagnosis and modeling of progression in Alzheimer's disease and aid risk prediction when integrated with multimodal data (53, 54).

This supports the rationale for including neuroimaging in multimodal data integration combined with digital phenotyping.

From a methodological perspective, the application of machine learning enables the handling of high-dimensional datasets and the identification of complex, non-linear relationships among clinical, digital, and biological variables. Systematic reviews on machine learning and wearable sensors highlight that such hybrid analytic frameworks can provide objective and reproducible measures of disease status in PD, although challenges remain in generalizability and real-world validation (51, 55).

The inclusion of lifestyle and environmental variables further strengthens the relevance of this protocol. While the effect of modifiable lifestyle factors on neurodegeneration is well recognized conceptually, empirical data integrating these variables with digital and biological markers remain less abundant, highlighting the potential of rAIdD to contribute novel evidence in this area.

Despite its strengths, the study also faces challenges. Multi-center harmonization of complex data streams, participant adherence to extended wearable monitoring, and the handling of heterogeneous real-world data are recognized methodological issues in large-scale digital health research. Moreover, although predictive models may achieve strong performance, clinical translation requires careful validation, external replication, and alignment with ethical and regulatory standards before implementation in routine care.

In conclusion, the rAIdD neurological study represents a robust and forward-looking protocol designed to advance predictive and personalized approaches in chronic neurological diseases by leveraging AI, wearable technologies, and multimodal data integration. This integrative strategy may yield clinically meaningful digital biomarkers and prognostic models, supporting more precise and patient-centered care.

## 4 Ethics and dissemination

This study has been reviewed and approved by the Local Ethical Committee of the coordinating center (AOU “G. Martino”–AO “Papardo”–A.S.P. Messina; reference number 0165421, 27/12/2023). Each participating institution obtained ethical clearance from its respective local Ethics Committee before patient enrollment. All participants (or their legal representatives, where applicable) provided written informed consent prior to participation, in accordance with the Declaration of Helsinki and the European General Data Protection Regulation (GDPR 2016/679).

Personal and clinical data will be handled in compliance with national and EU data protection laws, ensuring full anonymization and secure storage within the project's digital infrastructure. Data sharing across centers will follow controlled-access procedures to guarantee confidentiality and ethical use of participants' information.

Dissemination of the study results will be continuous throughout the project's duration and after its completion. Findings will be shared through multiple channels, including national and international scientific conferences, peer-reviewed open-access publications, and workshops involving clinicians, researchers, policymakers, and patient associations. Furthermore, periodic updates and project outputs will be made available through the rAIdD digital platform and institutional communication tools to ensure transparency and broad accessibility.

The research team is committed to promoting open science principles, facilitating knowledge transfer, and fostering collaborative networks within the national and international neurology community. Dissemination activities will aim to maximize the societal and clinical impact of the study, contributing to the advancement of predictive, preventive, and personalized approaches to chronic neurological diseases.

## Group member of the rAIdD Study Group

The members of the rAIdD Study Group are: Antonella Paola Agodi, Martina Barchitta, Daniela Giordano, Simone Palazzo, Aurora Zanghì, Carla Tortorella, Shalom Haggiag, Serena Ruggieri, Davide Dilena, Miloš Ajčević, Luca Gentile, Antonino Casile.
